# Determine Mesh Size through Monomer Mean-Square Displacement

**DOI:** 10.3390/polym11091405

**Published:** 2019-08-27

**Authors:** Ji-Xuan Hou

**Affiliations:** School of Physics, Southeast University, Nanjing 211189, China; jxhou@seu.edu.cn

**Keywords:** entangled polymer melt, tube theory, monomer mean-square displacement, mesh size

## Abstract

A dynamic method to determine the main parameter of the tube theory through monomer mean-square displacement is discussed in this paper. The tube step length can be measured from the intersection of the slope-$\frac{1}{2}$ line and the slope-$\frac{1}{4}$ line in log-log plot, and the tube diameter can be obtained by recording the time at which $g_{1}$ data start to leave the slope-$\frac{1}{2}$ regime. According to recent simulation data, the ratio of the tube step length to the tube diameter was found to be about 2 for different entangled polymer systems. Since measuring the tube diameter does not require $g_{1}$ data to reach the slope-$\frac{1}{4}$ regime, this could be the best way to find the entanglement length from microscopic consideration.

## 1. Introduction

Modern theories of polymer dynamics and rheology describe the universal aspects of the viscoelastic behavior based on the idea that molecular entanglements confine individual filaments to a one-dimensional, diffusive dynamics (reptation) in tube-like regions in space [1]. Entanglements are transient topological constraints arising from the restriction that the backbones of fluctuating chain molecules cannot cut through each other [2,3]. Since linear polymers are strongly interpenetrating, these constraints dominate the long-time dynamics of high molecular weight polymers, and entanglements strongly slow down the relaxation. A characteristic feature is subdiffusion regime $t^{1/4}$ in monomer mean-square displacement (MSD), which is even slower than the free three-dimensional Rouse motion $t^{1/2}$ [2,3].

Mesh size is one of most important quantities in tube theory. A quick method to predict mesh size is required and it should be easy to realize in computer simulations. Based on the concept of primitive path, the topological approach proposed by Everaers et al. is the so-called primitive path analysis (PPA), which can directly provide the statistical information of the primitive path mesh [4,5]. To obtain the precise results from PPA, one need to extrapolate the PPA results to infinite long chain [6], and this extrapolated result given by PPA has been verified by stress relaxation data [7]. An alternative method by using the monomer MSD was firstly proposed by Likhtman and McLeish [8]. This method is based on the scaling argument of the behaviors of MSD in different time regimes. Therefore, it is also subject to sufficient long chain system. Seeking a quick and easy method to determine the mesh size in medium-length entangled system is the aim of this paper.

The remainder of the paper is structured as follows. In Section 2, the difference between tube step length (TSL) and tube diameter (TD) is clarified. Section 3 is devoted to the calculation of the monomer MSD of entangled chain. Descriptions of determining the mesh size through MSD are presented in Section 4, and the ratio between these two quantities is given in Section 5. Finally, a brief summary is given in the last section.

## 2. Tube Step Length and Tube Diameter

In an entangled polymer melt, each polymer chain consists of *N* Kuhn beads and is confined in a tube formed by all other surrounding chains [2,9]. The center line of the tube is called primitive path (PP) which can be thought as a random Kuhn walk containing *Z* steps with the TSL *a*. The mean-square end-to-end distance of the tube should be equal to the mean-square end-to-end distance of the chain, $Za^{2}=\langle R_{ee}^{2}\rangle$. The entanglement length is defined as the number of monomers in a tube segment, $N_{e}=N/Z=Na^{2}/\langle R_{ee}^{2}\rangle$. $\tau_{R}$ and $\tau_{d}$ denote the Rouse time and the disentanglement time respectively. The entanglement time $\tau_{e}$ is defined as the Rouse time of the chain segment between entanglements, $\tau_{e}=\tau_{R}/Z^{2}$.

**Figure 1:**
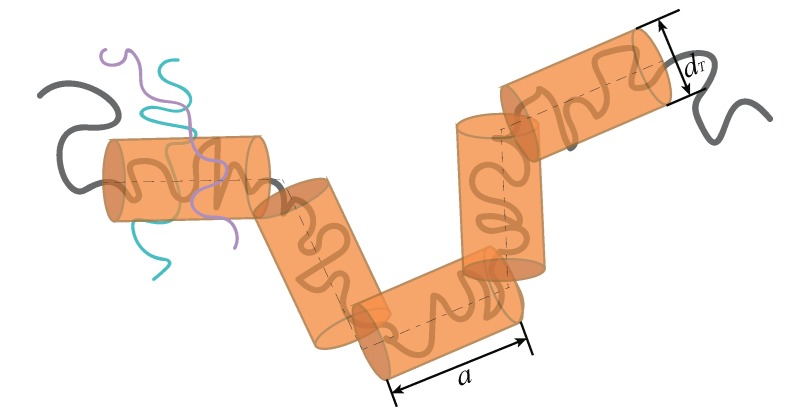
Schematic illustration of the tube. The black thick solid line and the thin dashing line represent the confined polymer chain and the primitive path, respectively.

The concept of TSL *a* is sometimes confused with TD $d_{T}$. TD specifies the range that a monomer can move perpendicularly to the tube. TSL and TD are two different concepts, although both share the same magnitude (see Figure 1). The linear rheological properties of an entangled melt are mainly determined by the TSL [2,8], while the mobility of nonsticky nanoparticles is governed by the ratio of the nanoparticle diameter $d_{P}$ to the TD [10,11,12,13,14,15].

## 3. Monomer Displacement in Entangled Linear Melts

The mean-square displacement (MSD) of a monomer in a melt is given by

(1) $$\begin{array}{c} g_{1}\left(t\right)\equiv \frac{1}{n_{c}N}\sum_{i=1}^{n_{c}}\sum_{j=1}^{N}\langle {\left[{\vec{r}}_{i,j}\left(t\right)-{\vec{r}}_{i,j}\left(0\right)\right]}^{2}\rangle , \end{array}$$

where $n_{c}$ and $\vec{r}$ denote the number of polymer chains and the position of the monomer, respectively. At a time smaller than the entanglement time $\tau_{e}$, $g_{1}\left(t\right)$ can be calculated by the three-dimensional Rouse model [16],

(2) $$\begin{array}{c} g_{1}\left(t\right)=\frac{2}{\pi^{3/2}}\langle R_{ee}^{2}\rangle {\left(\frac{t}{\tau_{R}}\right)}^{1/2}. \end{array}$$

At later time $\tau_{e}<t<\tau_{R}$, the motion of the Kuhn segment perpendicular to the tube is suppressed by the constraints, and the motion longitudinal to the primitive path can be calculated by the one-dimensional Rouse model with both chain ends stretched by an entropic force [2]. The curvilinear MSD is

(3) $$\begin{array}{c} g_{1\|}\left(t\right)\equiv \langle {\left(s_{n}\left(t\right)-s_{n}\left(0\right)\right)}^{2}\rangle =\frac{2}{3\pi^{3/2}}\langle R_{ee}^{2}\rangle {\left(\frac{t}{\tau_{R}}\right)}^{1/2}. \end{array}$$

$g_{1\|}\left(t\right)$ is just one third of $g_{1}\left(t\right)$ because only the one-dimensional longitudinal motion is allowed inside the tube in three-dimensional space. Thus, the motion perpendicular to the tube before the polymer chain meets the tube can be calculated by

(4) $$\begin{array}{c} g_{1⊥}\left(t\right)=g_{1}\left(t\right)-g_{1\|}\left(t\right)=\frac{2}{3}g_{1}\left(t\right)=\frac{4}{3\pi^{3/2}}\langle R_{ee}^{2}\rangle {\left(\frac{t}{\tau_{R}}\right)}^{1/2}. \end{array}$$

Since $s_{n}\left(t\right)-s_{n}\left(0\right)$ is longitudinal, the MSD in three-dimensional space is $\langle a|s_{n}\left(t\right)-s_{n}\left(0\right)|\rangle$. Therefore, at time $\tau_{e}<t<\tau_{R}$,

(5) $$\begin{array}{c} g_{1}\left(t\right)=\sqrt{\frac{2}{\pi }}a\sqrt{\langle {\left(s_{n}\left(t\right)-s_{n}\left(0\right)\right)}^{2}\rangle }=\frac{2}{\sqrt{3}\pi^{5/4}}a\sqrt{\langle R_{ee}^{2}\rangle }{\left(\frac{t}{\tau_{R}}\right)}^{1/4}. \end{array}$$

The prefactor $\sqrt{2/\pi }$ in the last equation appears since the distribution of segment displacement alone the tube is Gaussian.

## 4. Determination of Mesh Size

**Figure 2:**
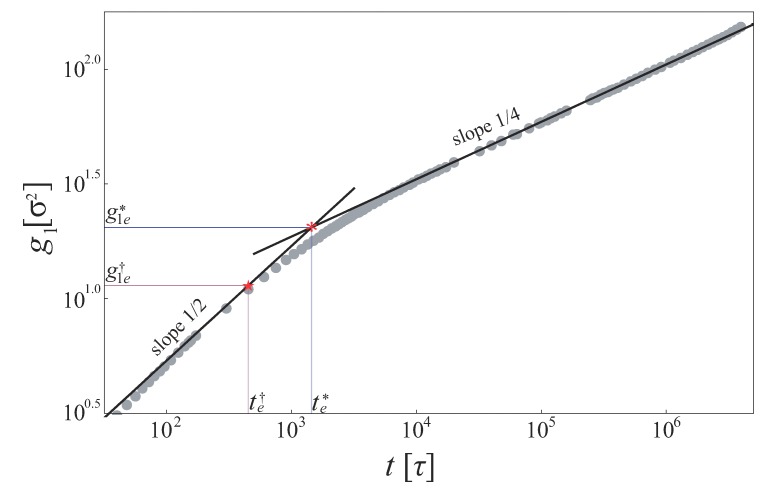
Monomer mean square displacement $g_{1}\left(t\right)$ of the Kremer–Grest model with $k_{\theta }=1.5$ from Reference [18]. The black solid lines are the best fits to the slope-$\frac{1}{2}$ regime and the slope-$\frac{1}{4}$ regime. The red asterisk is the intersection of two straight lines, and the red pentagram denotes the time at which the slope of $g_{1}\left(t\right)$ changes from 1/2.

In the log-log plot, the $g_{1}$ data can be fitted by straight lines in different time regimes ($t<\tau_{e}$ and $\tau_{e}<t<\tau_{R}$) (see Figure 2). One can obtain an intersecting point close to the entanglement time $\tau_{e}$ (the point marked by a red asterisk in Figure 2).

(6) $$\begin{array}{c} t_{e}^{*}=\frac{\pi }{9}\frac{a^{4}}{{\langle R_{ee}^{2}\rangle }^{2}}\tau_{R}=\frac{\pi }{9}\tau_{e}, \end{array}$$

(7) $$\begin{array}{c} g_{1e}^{*}=\frac{2}{3\pi }a^{2}. \end{array}$$

Therefore, TSL *a*, entanglement time $\tau_{e}$ and entanglement length $N_{e}$ can be obtain using this intersecting point,

(8) $$\begin{array}{c} a=\sqrt{\frac{3\pi }{2}g_{1e}^{*}}=\frac{\sqrt{3}}{\pi^{1/4}}\sqrt{\langle R_{ee}^{2}\rangle }{\left(\frac{t_{e}^{*}}{\tau_{R}}\right)}^{\frac{1}{4}} \end{array}$$

(9) $$\begin{array}{c} N_{e}=\frac{3\pi Ng_{1e}^{*}}{2\langle R_{ee}^{2}\rangle }. \end{array}$$

This dynamic method to determine TSL was firstly proposed by Likhtman and McLeish with a slightly different prefactor [8]. The entanglement length $N_{e}$ derived here is π/2≈1.57 times larger than the original prediction of reference [17].

Figure 2 shows the the most refined simulation data [18] of the Kremer–Grest model (KGM) [19] with the bond bending interactions parameter $k_{\theta }=1.5$ [18]. $t_{e}^{*}$ and $g_{1e}^{*}$ are estimated as $1.2\times 10^{3}\tau$ and $19\sigma^{2}$ using this method, where τ and σ are Lennard–Jones time unit and length unit, respectively. Using Equation (9), the entanglement length $N_{e}$ is estimated as 33, which is consistent with the PPA result (28).

There is a drawback, however: the slope-$\frac{1}{4}$ regime of the $g_{1}$ data is not easy to reach unless the simulated chains are extremely long. It is more common that the slop of $g_{1}$ data lies between 0.25 and 0.5 in the time regime of $\tau_{e}<t<\tau_{R}$. Stephanou et al. proposed an another method to estimate the TD by using $g_{1}$ data which do not need to reach the slope-$\frac{1}{4}$ regime [20,21,22]. Instead of finding the intersecting point between the slope-$\frac{1}{2}$ regime and the slope-$\frac{1}{4}$ regime, they recorded the time $t=t_{e}^{†}$ at which the $g_{1}$ data start to leave the initial slope-$\frac{1}{2}$ regime (see the point marked by a red pentagram in Figure 2). They supposed that, at time $t_{e}^{†}$, the monomer starts to feel the tube constraints, and the monomer displacement perpendicularly to the PP $g_{1⊥}\left(t_{e}^{†}\right)$ is comparable to the squared tube radius ${\left(d_{T}/2\right)}^{2}$. Thus,

(10) $$\begin{array}{c} \frac{d_{T}}{2}=\frac{\sqrt{\pi }}{2}\sqrt{g_{1⊥}\left(t_{e}^{†}\right)}=\frac{\sqrt{\pi }}{2}\sqrt{\frac{2}{3}g_{1e}^{†}}. \end{array}$$

The prefactor $\sqrt{\pi }/2$ above rises from the two-dimensional Gaussian distribution [23]. The TD becomes

(11) $$\begin{array}{c} d_{T}=\sqrt{\frac{2\pi }{3}g_{1e}^{†}}=\frac{2}{\sqrt{3}\pi^{1/4}}\sqrt{\langle R_{ee}^{2}\rangle }{\left(\frac{t_{e}^{†}}{\tau_{R}}\right)}^{\frac{1}{4}}. \end{array}$$

Note that the literature give a different result $d_{T}=2\sqrt{g_{1e}^{†}}$, which is about 1.38 times larger than Equation (11) [20,21,22].

## 5. The Ratio of TSL to TD

Based on the methods introduced in the last section, both TSL and TD can be estimated from monomer MSD data. Using Equation (8) and (11), the ratio of TSL to TD can be measured by

(12) $$\begin{array}{c} \frac{a}{d_{T}}=\frac{3}{2}{\left(\frac{g_{1e}^{*}}{g_{1e}^{†}}\right)}^{\frac{1}{2}}=\frac{3}{2}{\left(\frac{t_{1e}^{*}}{t_{1e}^{†}}\right)}^{\frac{1}{4}}. \end{array}$$

The parameters for linear polymer melts measured via MSD are given in Table 1. The measured results show the ratio $a/d_{T}≅2.0$ for different polymers.

The theoretical explanation for the ratio of 2 was given by Öttinger [30]. He adopted the Porod–Kratky wormlike chain model [31] for modeling entangled polymer chains. He showed that, for very long chains, the Kuhn length of the primitive chain turns out to be twice the TD (see Equation (47) in [30]).

It is well known that the PPA measurements (using Kröger’s Z algorithm [5] or CReTA algorithm [32]) show that the Kuhn length of the PP *a* is about twice as great as the mesh size of the PP network $d_{T}$ [6,32,33,34,35]. Everaers drew a particular analogy between the PP network and the phantom network, and he offered a physical interpretation of the ratio 2 [36]. Everaers’ argument is as follows: By treating the kinks or topological constraints of the PP network as the crosslinks of the phantom network model, the shear modulus is

(13) $$\begin{array}{c} G_{ph}=\left(1-\frac{2}{f}\right)\frac{\rho k_{B}T}{N_{e}^{\mathrm{topo}}} \end{array}$$

with the presence of crosslink fluctuations, where ρ, *f* and $N_{e}^{\mathrm{topo}}$ are the monomer density, arm number of the junction point and the number of monomers between the adjacent kinks, respectively. On the other hand, the melt plateau modulus is

(14) $$\begin{array}{c} G_{e}=\frac{\rho k_{B}T}{N_{e}^{\mathrm{PPKuhn}}} \end{array}$$

within the affine approximation, where $N_{e}^{\mathrm{PPKuhn}}$ is the number of monomers per PP Kuhn length. Since the kinks of the PP network can be thought as four-arm branch points (f=4) and $G_{ph}$ should be identical to $G_{e}$ in the rheological experiments, we have

(15) $$\begin{array}{c} \frac{a}{d_{T}}=\frac{N_{e}^{\mathrm{PPKuhn}}}{N_{e}^{\mathrm{topo}}}=2 \end{array}$$

by construction.

It is not easy to understand why the ratio of TSL to TD should be 2 due to the obscurity of the concept of tube. To get around the obscure picture of tube, Likhtman et al. proposed a simple model of a single entangled chain in a cubic lattice of line obstacles which is called grid model [37]. The grid model is intuitive and one could expect a perfect correspondence between this model and tube theory. One might anticipate that the TSL should be equal to the grid size. However, they found that the TSL measured by PPA in this model is also twice as large as the grid spacing for large grids.

As mentioned in Section 2, the mobility of non-sticky nanoparticles is governed by the ratio of nanoparticle diameter $d_{P}$ to TD $d_{T}$. Small particles ($d_{P}<d_{T}$) are only affected by the monomer motion, while large particles ($d_{P}>d_{T}$) are affected by the mesh formed by entangled polymers [10,11,12,13,14]. Thus, one can expect that the diffusion coefficient of the nanoparticles $D_{P}$ decreases as the increase of $d_{P}/d_{T}$ by

(16) $$\begin{array}{c} D_{P}∼\exp\left(-d_{P}/d_{T}\right). \end{array}$$

Recent simulation [15] shows that

(17) $$\begin{array}{c} D_{P}∼\exp\left(-cd_{P}/a\right), \end{array}$$

in which *c* is a fitting parameter and *a* is the TSL measured by PPA. The best-fit result is c=2.2±0.1. Therefore, comparing Equation (17) with Equation (16), the observation of this paper that $a/d_{T}=2$ is verified by the nanoparticle motion in entangled polymer melts.

If $a/d_{T}=2$ holds for all the situations, it becomes much easier to determine mesh size through monomer MSD. As discussed in Section 4, it is much easier to estimate TD $d_{T}$ than TSL *a*, because measuring *a* requires the slope-$\frac{1}{4}$ regime of MSD while measuring $d_{T}$ does not. Hence, one can measure TD $d_{T}$ by simulating short chain system (longer than $N_{e}$), and obtain TSL immediately by using the relation $a=2d_{T}$. This method to determine tube mesh size through MSD shown in this paper is more robust than other methods [8]. For example, one can also estimate the entanglement length by using the plateau modulus, $G_{e}=\rho k_{B}T/N_{e}$. However, this method has systematic error that comes from no-naffine deformation and the fluctuation of entanglement points [8]. Another method to determine the entanglement length is PPA. However, the results given by PPA are not reliable unless they are extrapolated to infinite long chain [6].

## 6. Summary

In this paper, we fully discuss a dynamic method to define the main parameter of the tube theory through monomer MSD $g_{1}$. The TSL can be measured from the intersection of the $t^{1/2}$ fitting line and the $t^{1/4}$ fitting line in log-log plot. The TD can be obtained by recording the time at which $g_{1}$ data start to leave the $t^{1/2}$ regime. Using this method, simulation data show that the TSL is twice as large as the TD.

## Figures and Tables

**Table 1:** Entanglement parameters for linear polymer melts measured via MSD. For time units, see the original references.

Ref.	Model or Material	$t_{e}^{†}$	$t_{e}^{*}$	$N_{e}$	$a/d_{T}$
[24]	KGM $k_{\theta }=0$	950τ	2950τ	85	2.0
[18]	KGM $k_{\theta }=1.5$	410τ	1200τ	33	2.0
[25]	KGM $k_{\theta }=2$	500τ	1610τ	28	2.0
[26]	KGM $k_{\theta }=2$	800τ	2290τ	35	2.0
[24]	KGM $k_{\theta }=2$	640τ	1900τ	34	2.0
[27]	Polybutadiene T=393 K	21,000$\tau_{s}$	72,400$\tau_{s}$		2.0
[28]	Continuous Model	10,000τ	31,500τ	43	2.0
[29]	Coarse–Grained Model	$5.5\times 10^{-6}\tau$	$1.85\times 10^{-5}\tau$		2.0
